# Supplementary material for: Is There a Bidirectional Association between Polycystic Ovarian Syndrome and Periodontitis? A Systematic Review and Meta-analysis
Source: J Clin Med. 2020 Jun 23;9(6):1961. doi: 10.3390/jcm9061961 (PMC7355910; doi:10.3390/jcm9061961)
Supplement: Supplementary file 1 [file jcm-09-01961-s001.pdf]

Journal of
Clinical Medicine

MDPI

Review

Is There a Bidirectional Association between Polycystic Ovarian Syndrome and Periodontitis? A Systematic Review and Meta-analysis

Vanessa Machado [1,*], Cláudia Escalda [1], Luís Proença [2], José João Mendes [1] and João Botelho [1]

1 Clinical Research Unit (CRU), Centro de Investigação Interdisciplinar Egas Moniz (CiiEM), Instituto Universitário Egas Moniz (IUEM), 2829-511 Caparica. Portugal; claudia.escalda4@gmail.com (C.E.); jmendes@egasmoniz.edu.pt (J.J.M.); jbotelho@egasmoniz.edu.pt (J.B.)
2 Quantitative Methods for Health Research (MQIS), Centro de Investigação Interdisciplinar Egas Moniz (CiiEM), Instituto Universitário Egas Moniz (IUEM), 2829-511 Caparica, Portugal; lproenca@egasmoniz.edu.pt
* Correspondence: vmachado@egasmoniz.edu.pt

Supplementary material

Table 1. List of excluded articles

	Number	Reference	Reason for exclusion
2019	1	Varghese, Arun, A. Julie Christy, B. Pratheeba, P. Saraswathy, and Sumathy Ravi. "Salivary Interleukin-6 Levels among Polycystic Ovary Syndrome (Pcos) Patients with and Without Chronic Periodontitis-A Comparitive Study." International Journal of Innovative Research in Medical Science 4, no. 09 (2019): 534-to.	Unrelated group
2017	2	Deepti, Tewari S, Narula SC, Singhal SR, Sharma RK. Effect of Non-Surgical Periodontal Therapy Along With Myo-Inositol on High-Sensitivity C-Reactive Protein and Insulin Resistance in Women With Polycystic Ovary Syndrome and Chronic Periodontitis: A Randomized Controlled Trial. J Periodontol. 2017;88(10):999–1011. doi:10.1902/jop.2017.170121	No control group
2017	3	Akcalı A, Bostanci N, Özçaka Ö, et al. Gingival Inflammation and Salivary or Serum Granulocyte-Secreted Enzymes in Patients With Polycystic Ovary Syndrome. J Periodontol. 2017;88(11):1145–1152. doi:10.1902/jop.2017.170043	No periodontitis group
2017	4	Nair SD, Varma S, Suragimath G, et al. Prevalence of periodontal disease in women with polycystic ovary syndrome- A comparative descriptive study. J. Evolution Med. Dent. Sci. 2017;6(65):4733-4736, DOI:10.14260/Jemds/2017/1025	Sample size bias
2015	5	Akcalı, A., Bostanci, N., Özçaka, Ö., Öztürk-Ceyhan, B., Gümüş, P., Tervahartiala, T., Husu, H., Buduneli, N., Sorsa, T., & Belibasakis, G. N. (2015). Elevated matrix metalloproteinase-8 in saliva and serum in polycystic ovary syndrome and association with gingival inflammation. Innate	No periodontitis group

		immunity, 21(6), 619–625. https://doi.org/10.1177/1753425915572172	
2014	6	Asnani, K. P., Hingorani, D., Kheur, S., Deshmukh, V., & Romanos, G. E. (2014). Expression of nuclear receptors of gingiva in polycystic ovarian syndrome: a preliminary case study. Australian dental journal, 59(2), 252–257. https://doi.org/10.1111/adj.12176	Case report
2012	7	Özçaka, Ö., Ceyhan, B. Ö., Akcali, A., Biçakci, N., Lappin, D. F., & Buduneli, N. (2012). Is there an interaction between polycystic ovary syndrome and gingival inflammation?. Journal of periodontology, 83(12), 1529–1537. https://doi.org/10.1902/jop.2012.110588	Repeated sample

Table 2. Case-control studies bias assessment using the Newcastle-Ottawa Scale (NOS)

**Study.**	**SELECTION**				**COMPARABILITY**	**EXPOSURE**			**RoB Score**
	**Is the case definition adequate?**	**Representativeness of the cases?**	**Selection of controls?**	**Definition of controls?**	**Comparability of cases and controls of design or analysis?**	**Ascertainment of exposure?**	**Same method of ascertainment for cases**	**Non-response rate?**	
Dursun et al. 2011	a	b	c	a	a/b	a	a	a	7
Ozçaka et al. 2013	a	a	a	b	a/b	a	a	a	8
Porwal et al. 2014	a	a	a	a	a/b	a	a	a	9
Akcali et al. 2014	a	b	a	a	a/b	a	a	a	8
Rahiminejad et al. 2015	a	a	a	b	a/b	a	a	a	8
Hameed et al. 2017	a	b	a	a	a/b	a	a	a	8
Najah et al. 2017	a	b	a	a	a/b	a	a	a	8
Saglam et al. 2018	a	b	a	a	a/b	a	a	a	8
Tong et al. 2019	b	a	a	b	a/b	a	a	a	7
Varadan et al. 2019	a	a	a	a	a/b	a	a	a	9
Isik et al. 2020	a	a	a	a	a/b	a	a	a	9
Saljoughi et al. 2020	a	a	a	a	a/b	a	a	a	9
